# к Opioids inhibit tumor angiogenesis by suppressing VEGF signaling

**DOI:** 10.1038/srep03213

**Published:** 2013-11-14

**Authors:** Kohei Yamamizu, Sadayoshi Furuta, Yusuke Hamada, Akira Yamashita, Naoko Kuzumaki, Michiko Narita, Kento Doi, Shiori Katayama, Hiroshi Nagase, Jun K. Yamashita, Minoru Narita

**Affiliations:** 1 Laboratory of Stem Cell Differentiation, Stem Cell Research Center, Institute for Frontier Medical Sciences, Kyoto University, Kyoto, Japan; 2 Department of Cell Growth and Differentiation, Center for iPS Cell Research and Application (CiRA), Kyoto University, Kyoto, Japan; 3 Laboratory of Genetics, National Institute on Aging, National Institutes of Health, Baltimore, MD, USA; 4 Department of Pharmacology, Hoshi University School of Pharmacy and Pharmaceutical Sciences, Tokyo, Japan; 5 Department of Physiology, Keio University, School of Medicine, Tokyo, Japan; 6 Department of Medicinal Chemistry, International Institute for Integrative Sleep Medicine (IIIS), University of Tsukuba, Tsukuba, Japan; 7 These authors contributed equally to this work.

## Abstract

Opioids are effective analgesics for the management of moderate to severe cancer pain. Here we show that κ opioid receptor (KOR) agonists act as anti-angiogenic factors in tumors. Treatment with KOR agonists, U50,488H and TRK820, significantly inhibited human umbilical vein endothelial cell (HUVEC) migration and tube formation by suppressing VEGFR2 expression. In contrast, treatment with a μ opioid receptor agonist, DAMGO, or a δ opioid receptor agonist, SNC80, did not prevent angiogenesis in HUVECs. Lewis lung carcinoma (LLC) or B16 melanoma grafted in KOR knockout mice showed increased proliferation and remarkably enhanced tumor angiogenesis compared with those in wild type mice. On the other hand, repeated intraperitoneal injection of TRK820 (0.1–10 μg/kg, b.i.d.) significantly inhibited tumor growth by suppressing tumor angiogenesis. These findings indicate that KOR agonists play an important role in tumor angiogenesis and this knowledge could lead to a novel strategy for cancer therapy.

Angiogenesis is a key event in vascular development and organogenesis in the embryo, as well as in physiological tissue remodeling and pathologic disorders, particularly tumorigenesis and metastasis. Tumor angiogenesis is required for tumor progression, with the intake of nutrients and oxygen as well as the excretion of metabolic wastes and carbon dioxide^1^,^2^. The balance between endogenous angiogenesis activators and inhibitors critically maintains a normally quiescent vasculature to sustain homeostasis. Disruption of the balance between angiogenesis activators and inhibitors causes pathogenic angiogenesis, and especially in tumors several activators such as vascular endothelial growth factor (VEGF) are highly expressed in the tumor microenvironment and strongly induce tumor angiogenesis^3^. Therefore, restoration of the balance between activators and inhibitors for angiogenesis is a critical treatment strategy for tumors.

VEGF plays a pivotal role in neovascularization in the embryo as well as in the adult mainly through VEGF receptor 2 (VEGFR2; also known as KDR in human and Flk1 in mouse). Expression of the VEGF gene has been shown to be upregulated by hypoxia and oncogene signaling such as Ras and Myc in cancer cells, which would lead to the formation of vasculature and the proliferation of tumors^4^,^5^,^6^. Consequently, numerous drugs have been developed to inhibit tumor angiogenesis by suppressing VEGF signaling. Furthermore, the blocking of antibodies against neuropilin1, a VEGF co-receptor, additively prevented the progression of tumors when combined with anti-VEGF drugs^7^. In clinical medicine, bevacizumab, a humanized monoclonal antibody that is specific for human VEGF, is the first anti-angiogenic agent for the treatment of colorectal cancer, renal cell cancer, non-small cell lung cancer, and glioblastoma^8^. Although therapies that inhibit tumor angiogenesis have been highly successful for tumor therapy, most patients eventually acquire resistance to anti-angiogenic therapy. Thus, we must identify novel targets for anti-angiogenic therapeutics to achieve the continuous inhibition of angiogenesis for tumor therapy.

Three opioid receptors, μ, δ, and κ (MOR, DOR, and KOR), regulate physiological functions, including pain regulation, emotional tone, and reward circuitry^9^. Opioid analgesics such as morphine, a MOR agonist, have been broadly applied to relieve pain from all types of cancer. A recent study showed that morphine suppresses tumor angiogenesis through the inhibition of hypoxia-inducible transcription factors, which enhances the expression of VEGF and VEGF receptors^10^. However, the effect of morphine on tumor growth is still controversial. Independent studies have shown that morphine can either decrease or increase tumor growth in mice^11^,^12^. More recently, we showed that κ opioid peptides acted as novel anti-angiogenic modulators through suppressing the expression of VEGF receptors, VEGFR2 and Neuropilin1, during vascular differentiation via inhibition of cAMP/PKA signaling^13^. In this study, we first demonstrated that KOR agonists have a potential as anti-tumor angiogenic modulators by inhibiting VEGFR2 expression. These findings support a novel strategy for tumor therapy as well as the relief of cancer pain.

## Results

### KOR agonists inhibit the migration of and capillary structure formation by endothelial cells

**Figure 1:**
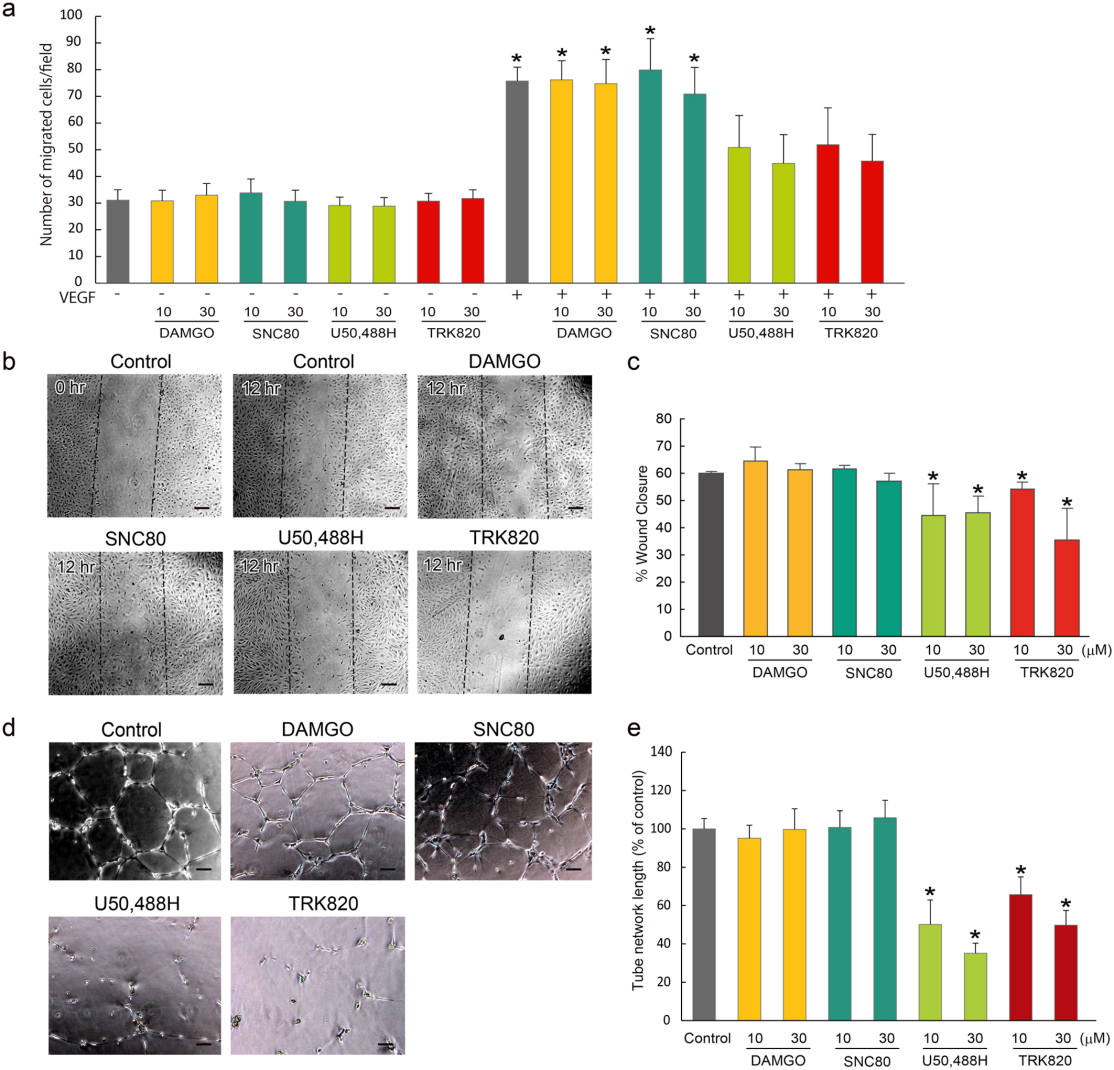
Inhibitory effects of KOR agonists, U50,488H and TRK820, on HUVEC migration and tube formation. (a) The boyden chamber assay. Inhibition of VEGF-induced chemotaxis was assessed after including DAMGO (10, 30 μM), SNC80 (10, 30 μM), U50,488H (10, 30 μM), or TRK820 (10, 30 μM) (n = 3, *p < 0.05 vs. Control). (b) The wound-healing assay. HUVECs were plated, scratched and then incubated with DAMGO (10, 30 μM), SNC80 (10, 30 μM), U50,488H (10, 30 μM) or TRK820 (10, 30 μM) as indicated. Scale bars: 200 μm. (c) Quantitative evaluation of the effect of opioid receptor agonists on HUVEC wound-healing assay. Three independent experiments are shown (n = 3, *p < 0.05 vs. Control). (d) HUVEC tube formation assay. Representative photographs of vasculature with DAMGO (10 μM), SNC80 (10 μM), U50,488H (10 μM) or TRK820 (10 μM). Scale bars: 200 μm. (e) Quantitative evaluation of the effect of opioid receptor agonists on HUVEC tube formation assay. Three independent experiments are shown (n = 3, *p < 0.05 vs. Control).

Endothelial cell (EC) migration is a critical step to form new blood vessels during angiogenesis. To investigate the roles of opioids in EC migration, we performed a boyden chamber assay and a wound-healing assay with HUVECs. Treatment with VEGF significantly increased migrated EC cells with a boyden chamber assay. Treatment with KOR agonists, U50,488H or TRK820, together with VEGF decreased migrated EC cells (Fig. 1a). In contrast, neither the MOR agonist DAMGO nor the DOR agonist SNC80 inhibited EC migration. The consistent results was shown that treatment with U50,488H or TRK820 significantly inhibited EC migration with a wound-healing assay (Fig. 1b, c). These effects on EC migration were inhibited by treatment with nor-binaltorphimine (BNI), a selective KOR antagonist, or knockdown of KOR with siRNA (Supplemental Fig. 1a–d). Furthermore, we examined HUVEC tube formation using a 2-dimensioned matrigel assay. Both U50,488H and TRK820, but not DAMGO or SNC80, dramatically inhibited HUVEC tube formation (Fig. 1d, e). These effects on tube formation were inhibited by knockdown of KOR with siRNA (Supplemental Fig. 1e). These findings indicate that KOR signaling could regulate angiogenesis *in vitro*.

### KOR agonists suppress VEGFR2 expression in endothelial cells

**Figure 2:**
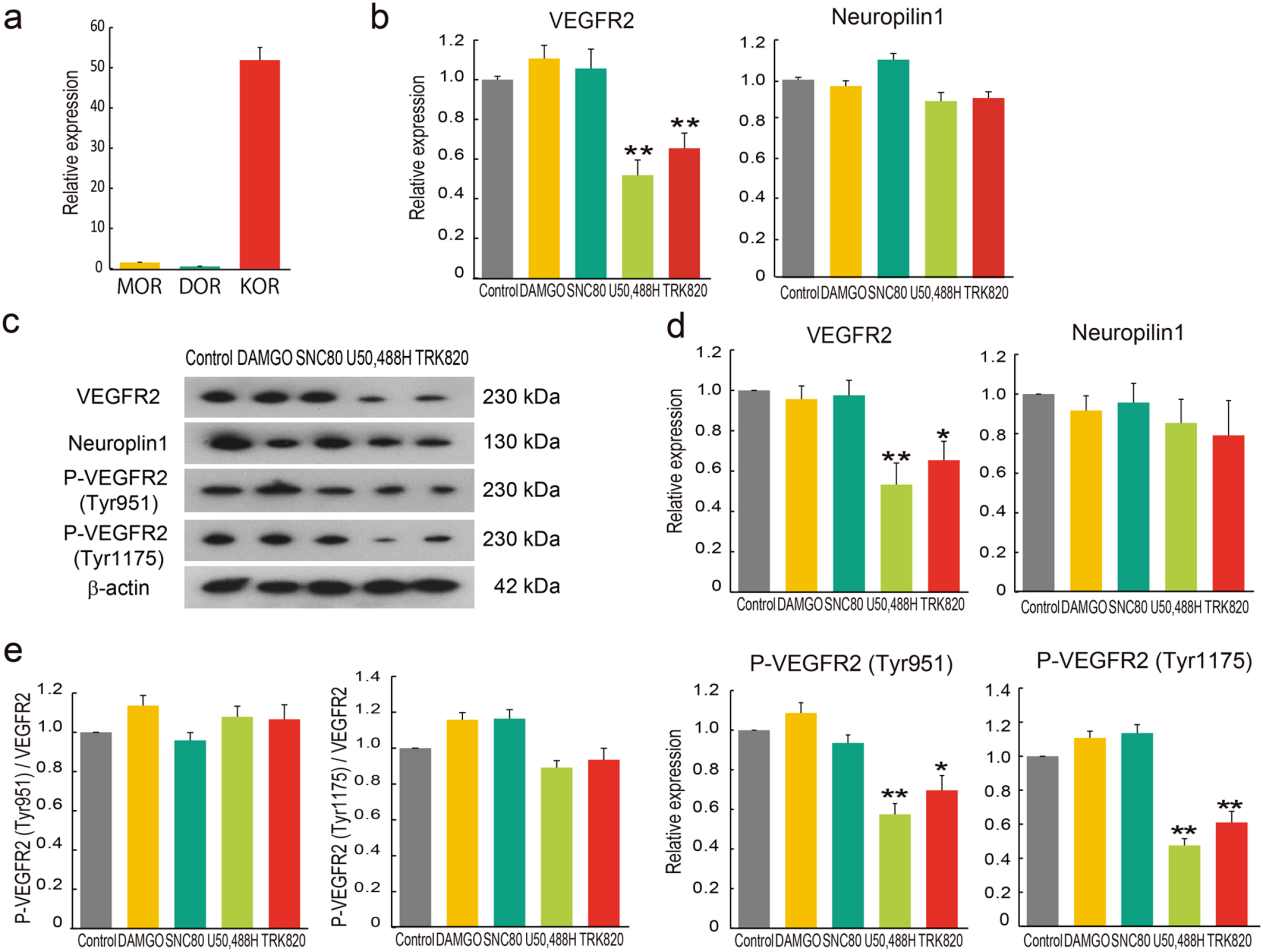
Inhibitory effects of KOR agonists, U50,488H and TRK820, on VEGFR2 expression in HUVECs. (a) qPCR showing mRNA expression of MOR, DOR, and KOR in HUVECs. (b) qPCR showing mRNA expression of VEGFR2 and Neuropilin1 after 24 hr culture with DAMGO (10 μM), SNC80 (10 μM), U50,488H (10 μM) or TRK820 (10 μM). (c) Western blotting of VEGFR2, Neuroplin1, VEGFR2 phospho-Tyr951, VEGFR2 phospho-Tyr1175, or β-actin after 24 hr culture with DAMGO (10 μM), SNC80 (10 μM), U50,488H (10 μM) or TRK820 (10 μM). These gels have been run under the same experimental conditions (see Methods). These cropped blots are used in the main figures and these full-length blots are included in the supplementary information (Supplementary Fig. 2). (d) Quantitative evaluation of the effect of opioid receptor agonists by western blotting. Normalization of expression of VEGF, Neuroplin1, VEGFR2 phospho-Tyr951, or VEGFR2 phospho-Tyr1175 is addressed by expression of β-actin. Three independent experiments are shown (n = 3, **p < 0.01, *p < 0.05 vs. Control). (e) Quantitative evaluation of the effect of opioid receptor agonists by western blotting. Normalization of expression of VEGFR2 phospho-Tyr951 or VEGFR2 phospho-Tyr1175 is addressed by expression of VEGFR2.

As shown in Fig. 2a, KOR was highly expressed in HUVECs, whereas MOR and DOR were weakly expressed. Opioid receptors transduce signals through G inhibitory protein to inhibit abenylyl cyclase and subsequently decrease cAMP production and inactivate PKA. We previously revealed that KOR agonists inhibit EC differentiation from ES cells through suppression of VEGFR2 and Neuroplin1 expressions via inhibition of cAMP/PKA signaling^13^. Therefore, we examined VEGF receptor expression after the activation of opioid receptors. Similar, but not same to previous results^13^, in HUVECs, VEGFR2 protein, but not Neuropilin1, was significantly down-regulated by the addition of U50,488H or TRK820 (Fig. 2b, 2c, supplementary Fig. 2). Neither DAMGO nor SNC80 affected VEGFR expression in HUVECs. We further investigated the phosphorylation of VEGFR2 after the activation of opioid receptors. VEGFR2 phosphorylation at Tyrosine 951 (Tyr951) and 1175 (Tyr1175) mainly activates downstream signals and leads to EC migration and proliferation, thereby forming vasculature^14^. In this study, VEGFR2 phosphorylation at both Tyr951 and Tyr1175 was specifically suppressed by treatment with U50,488H or TRK820, but these suppression of VEGFR2 phosphorylation are depend on protein expression of VEGFR2 (Fig. 2b–d). These results indicate that KOR signaling specifically regulates VEGFR2 expression in ECs.

### Loss of KOR increases tumor angiogenesis

**Figure 3:**
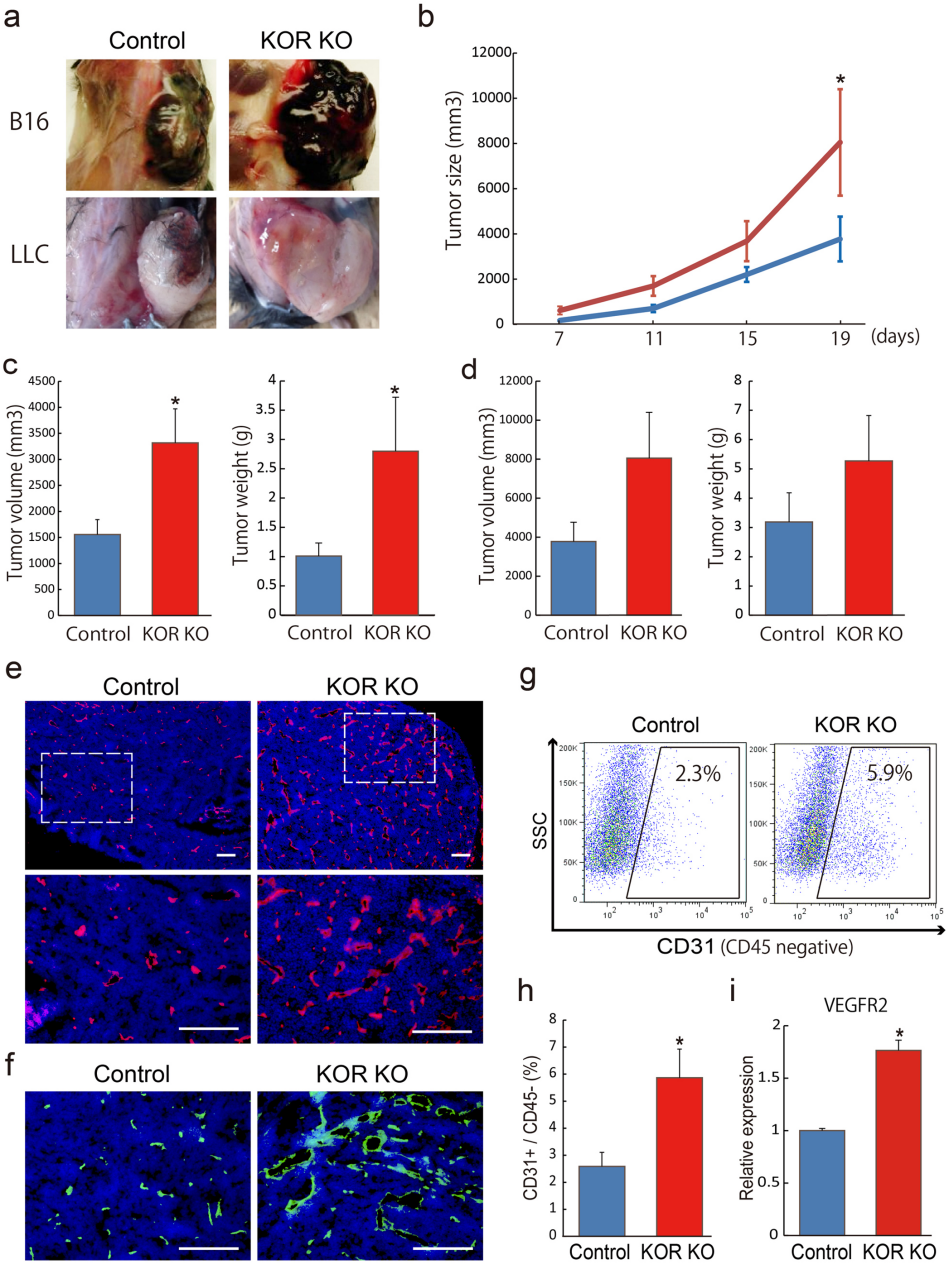
Increase of tumor angiogenesis in graft KOR KO mice. (a) Typical example of B16 or LLC-bearing control (left) or KOR KO (right) mice. (b) Quantitative analysis of tumor size between control (n = 5) and KOR KO (n = 6) mice at 7, 11, 15, 19 days (*p < 0.05 vs. Control). (c) The tumor volume and the tumor weight between B16 grafted-control (n = 10) and -KOR KO (n = 9) mice at 19 days (*p < 0.05 vs. Control). (d) The tumor volume and the tumor weight between LLC grafted-control (n = 5) and -KOR KO (n = 6) mice at 19 days. (e) Fluorescent staining for CD31 (red) at 19 days. Nuclei are stained with DAPI (blue). Left panel, Control. Right panel, KOR KO. Scale bars: 200 μm. (f) Fluorescent staining for VE-cadherin (green) at 19 days. Nuclei are stained with DAPI (blue). Left panel, Control. Right panel, KOR KO. Scale bars: 200 μm. (g) Flow cytometry. X-axis: CD31 (CD45 negative), Y-axis: SSC. Percentages of CD31^+^/CD45^−^ ECs among tumors-dissociated cells are indicated. (h) Quantitative evaluation on CD31^+^/CD45^−^ EC population in tumors by FACS. Percentages of CD31^+^/CD45^−^ cell population among tumors-dissociated cells. Tumors transplanted Control (n = 4) or KOR KO (n = 4) mice are shown (*p < 0.05 vs. Control). (i) qPCR showing mRNA expression of VEGFR2 in purified ECs from LLC grafted in control mice or KOR KO mice at 19 days after tumor transplantation.

We confirmed the effects of KOR on tumor angiogenesis *in vivo* using a KOR knock out (KO) animal model. We previously demonstrated that KOR KO mice increased vascular formation in early stages (from embryonic day-8.75 to embryonic day-11.5) of vascular development, but KOR KO mice are viable by diminishing abnormal vascular formation from late stages of vascular development^13^. These results suggest that the KOR system could control neo-vascular formation *in vivo*. To investigate whether KORs are involved in tumor angiogenesis, we subcutaneously implanted Lewis lung carcinoma (LLC) or B16 melanoma into KOR KO mice and control mice. Interestingly, the tumor volume and the tumor weight of both LLC and B16 in KOR KO mice were significantly greater than that in control mice at 19 days after transplantation (Fig. 3a–d). To assess whether a loss of KOR signaling increases angiogenesis in tumors, we performed immunostaining and FACS analysis with tumors. LLC grafted in KOR KO mice increased ECs as shown the endothelial markers, CD31 and VE-cadherin (Fig. 3e, f). Furthermore, FACS analysis showed that LLC grafted in KOR KO mice showed significantly enhanced CD31 positive angiogenesis compared to that in control mice (percentage of CD31^+^/CD45^−^ ECs; 2.59 ± 0.52 in control mice vs. 5.89 ± 1.06 in KOR KO mice, n = 4 (independent mice), *p < 0.05, Fig. 3g, h). We confirmed the expression of opioid receptors and VEGFR2 in CD31-positive ECs purified from tumors. Purified ECs from LLC grafted in control mice highly expressed KOR, but not MOR or DOR (Supplemental Fig. 3). ECs from LLC grafted in KOR KO mice showed a significant increase in VEGFR2 compared with ECs from LLC grafted in control mice (Fig. 3i). These results suggest that KOR could play a critical role as anti-angiogenic mediators in tumors.

### KOR agonists inhibit tumor angiogenesis and tumor growth

**Figure 4:**
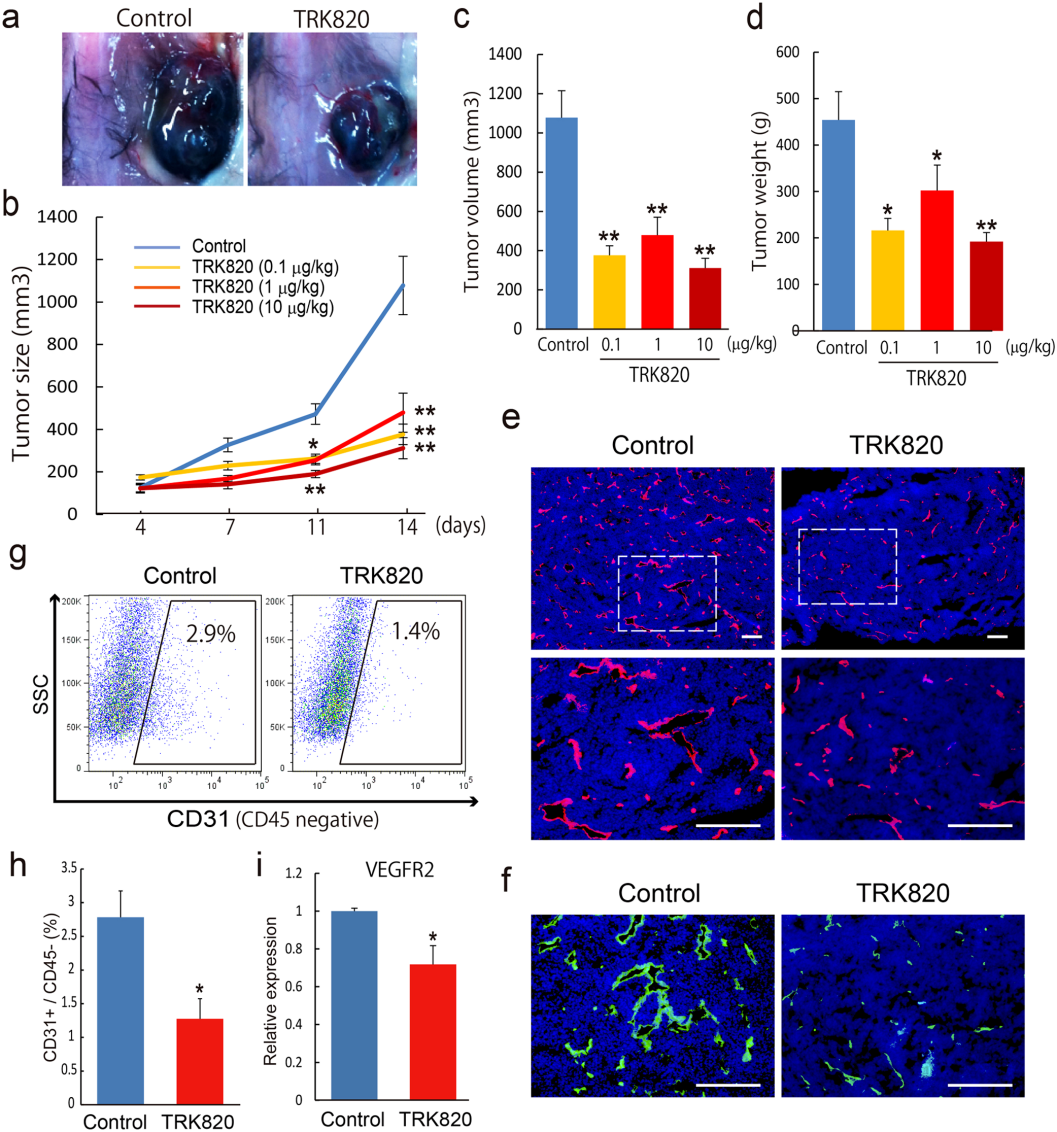
Suppression of tumor growth by a KOR agonist, TRK820 in graft mice. (a) Typical example of B16 -bearing control (left) or TRK-820 treated (right) mice. (b) Quantitative analysis of tumor size among PBS-treated (n = 16) and TRK820 (0.1 μg/kg (n = 9), 1 μg/kg (n = 17), 10 μg/kg (n = 9)-treated mice at 4, 7, 11, 14 days after tumor transplantation (**p < 0.01, *p < 0.05 vs. Control). (c, d) Quantitative analysis of tumor volume and tumor weight among PBS-treated (n = 16) and TRK820 (0.1 μg/kg (n = 9), 1 μg/kg (n = 17), 10 μg/kg (n = 9)-treated mice at 14 days after tumor transplantation (**p < 0.01, *p < 0.05 vs. Control). (e) Fluorescent staining for CD31 (red) at 14 days after tumor transplantation. Nuclei are stained with DAPI (blue). Left panel, PBS treated. Right panel, TRK820 (1 mg/kg)-treated. Scale bars: 200 μm. (f) Fluorescent staining for VE-cadherin (green) at 14 days after tumor transplantation. Nuclei are stained with DAPI (blue). Left panel, PBS treated. Right panel, TRK820 (1 mg/kg)-treated. Scale bars: 200 μm. (g) Flow cytometry. X-axis: CD31 (CD45 negative), Y-axis: SSC. Percentages of CD31^+^/CD45^−^ ECs among tumors-dissociated cells are indicated. (h) Quantitative evaluation on CD31^+^/CD45^−^ EC population in tumors by FACS. Percentages of CD31^+^/CD45^−^ cell population among tumors-dissociated cells. Tumors transplanted PBS-treated (n = 4) and TRK820 (1 μg/kg)-treated (n = 4) mice are shown (*p < 0.05 vs. Control). (i) qPCR showing mRNA expression of VEGFR2 in purified ECs from B16 grafted PBS-treated (n = 4) and TRK820 (1 μg/kg (n = 4))-treated mice at 14 days after tumor transplantation.

Since κ opioids could act as inhibitors of tumor angiogenesis, we considered the possibility of tumor therapy with KOR agonists. Using a mouse B16 graft model, we examined the inhibitory function of tumor angiogenesis by TRK820. The repeated intraperitoneal injection of TRK820 (0.1, 1, or 10 μg/kg, b.i.d.) every 12 hr significantly decreased the tumor size from 11 days to 14 days after transplantation (Fig. 4a, b). The tumor volume and tumor weight in TRK820-treated mice were much less than those in control mice at 7 days and 14 days after transplantation (Fig. 4c, 4d, supplementary Fig. 4a). In contrast, TRK820 had no inhibitory effects in B16 grafted in KOR KO mice, indicating that TRK820 specifically induced the inhibition of tumor angiogenesis and tumor growth through KOR receptors (Supplemental Fig. 5). We performed the immunostaining and FACS analysis to examine tumor angiogenesis. Treatment with TRK820 remarkably suppressed the both CD31 and VE-cadherin positive tumor angiogenesis at 7 days and 14 days after transplantation (Fig. 4e, 4f, supplementary Fig. 4b). Moreover, CD31 positive ECs in B16 grafted in TRK820-treated mice were significantly decreased compared with those control mice at 14 days after transplantation (percentage of CD31^+^/CD45^−^ ECs; 2.78 ± 0.39 in control mice vs. 1.28 ± 0.30 in KOR KO mice, n = 4 (independent mice), *p < 0.05, Fig. 4e). Purified ECs from B16 grafted in TRK820-treated mice showed a significant decrease in VEGFR2 compared with ECs from B16 grafted in control mice (Fig. 4i). Taken together, these results suggest that the KOR agonist TRK820 could inhibit tumor angiogenesis through inhibition of VEGFR2 expression, thereby suppressing tumor growth.

## Discussion

Endogenous angiogenesis inhibitors function as physiologic modulators that control angiogenesis during tissue remodeling as well as tumor formation^15^. In this study, we demonstrated that the κ opioid system acts the novel endogenous angiogenesis inhibitor in tumor. Zabrenetzky et al revealed that expression of thrombospondin1 (TSP1) which is the first protein to be recognized as an endogenous angiogenesis inhibitor is inversely correlated with malignant progression in melanoma, lung and breast carcinoma^16^. Suppression of TSP1 augmented tumor angiogenesis through matrix metalloprotease 9 production and enhancement of VEGFR2 signaling^16^. In contrast, TSP1 overexpression resulted in delayed tumor growth by inhibition tumor angiogenesis^17^. Present data showed similar results that a loss of KOR function, through the use of KO mice, remarkably increased tumor angiogenesis and tumor growth. Conversely, treatment with KOR agonists prevented tumor growth.

To date, approximately 30 endogenous angiogenesis inhibitors have been identified^18^. Many endogenous angiogenesis inhibitors including TSP1 are fragments of naturally occurring extracellular matrix and basement membrane proteins^19^. Although novel anti-angiogenesis drugs that target other molecules on tumors are needed to complement existing therapies, it is difficult to accurately control their expression and to apply them in clinical medicine. In contrast, opioids have been used clinically as effective analgesics for a long time. Our findings showed that the KOR system could act directly on tumor angiogenesis to suppress VEGFR2 expression; to our knowledge, this is the first report of an endogenous angiogenesis inhibitor of a ligand-receptor system that suppresses the expression of VEGF receptor. Thus, the molecular regulation of KOR systems may be a potential therapeutic strategy for cancers.

Interestingly, the KOR agonist TRK820 (nalfurafine), which has been clinically approved in Japan for use in hemodialysis-related uremic pruritus, could be useful for tumor therapy by suppressing tumor angiogenesis, and thus could offer therapeutic benefits beyond the relief of cancer pain. However, patients develop tolerance to opioid receptor agonists including TRK820 through repeated use^20^. Although a low dose (0.1–10 μg/kg, b.i.d.) of TRK820, which is effective for managing itching and pain in mice, significantly inhibited tumor angiogenesis and tumor growth at 7 and 14 days, an extremely high dose (150 μg/kg) had no significant effect on tumor growth (data not shown). These results suggest that continuous treatment with KOR agonists might lead to the development of tolerance to their anti-angiogenic effects on tumors. Therefore, more precise and careful observations are required to establish tumor therapies with KOR agonists. Nevertheless, a better understanding of the anti-angiogenic effects of κ opioids and the ability to manipulate the ligand-receptor system of opioids in tumor angiogenesis should greatly contribute to basic vascular biology as well as to applied cancer therapy beyond the relief of cancer pain.

## Methods

### Cell culture

Primary human umbilical vascular endothelial cells (HUVECs) were purchased from Lonza. HUVECs were cultured in EC growth medium (EGM-2 BulletKit, Lonza) supplemented with 2% fetal bovine serum (FBS). Lewis lung carcinoma (LLC) and B16 melanoma were kind gifts from Dr. Shibuya and Dr. Muramatsu. LLC or B16 cells were cultured in DMEM supplemented with 10% FBS and 1% antibiotics. Various reagents were occasionally added to the HUVEC culture, including [D-Ala2, N-Me-Phe4, Gly5-ol]-enkephalin (DAMGO) (Sigma), [(+)-4-[(aR)-a-((2S,5R)-4-allyl-2,5-dimethyl-1-piperazinyl)-3-methoxybenzyl] -N,N-diethylbenzamide] (SNC80) (Tocris Cookson Ltd, Ballwin, MO), (–)-trans-(1S,2S)-U-50488 hydrochloride (U50,488H) (Research Biochemicals International, Natick, MA), and 17-cyclopropylmethyl-3,14β-dihydroxy-4,5α-epoxy-6β-[N-methyl-trans-3-(3-furyl) acrylamido]morphinan hydrochloride (TRK-820) (TORAY, Tokyo, Japan).

### Migration and tube formation assay

In the boyden chamber assay, HUVECs were serum-starved with EBM2 medium (0.1% FBS, no growth factor) (Lonza) for 12 hours, and HUVEC (10^5^ cells per well) were seeded into the upper well of a boyden chamber system (Corning, New York, NY) on polyethylene terephthalate membrane with 8-μm pores. Human recombinant VEGF165 (R&D Systems, Minneapolis, MN) was added as a chemo-attractant into the lower well at 20 ng/ml. Inhibition of VEGF-induced chemotaxis was assessed after including DAMGO, SNC80, U50,488H, or TRK-820 at relevant doses. Migration through the membrane was determined after 4 h of incubation at 37°C by fixing, staining with hematoxylin and eosin, and counting the migrated cells in five random fields at 100 × magnification.

In the wound-healing assay, HUVECs that had grown to confluence in 24-well culture plates were serum-starved with EBM2 medium (0.1% FBS, no growth factor) (Lonza) for 12 hours, and a portion of the cell monolayer was then scraped away with a P200 pipette tip. The remaining cells were gently washed with medium and incubated for 16 hours in EGM-2 BulletKit. EC migration from the edge of the injured monolayer was quantified by measuring the distance between the wound edges, at three random positions in one visual field, before and after incubation with the use of a computer-assisted microscope (Leica) using Axiovision (Zeiss) and Image J (NIH).

In the tube formation assay, HUVECs (1.5 × 10^4^) were cultured in a 24-well plate (Gibco) coated with 150 μl Matrigel Basement Membrane Matrix GFR (BD Biosciences). These experiments were performed with various reagents such as DAMGO, SNC80, U50,488H or TRK-820. EC tube length was quantified at five random positions by Image J (NIH).

### Western blotting

Western blotting was performed as described previously^21^,^22^. Briefly, HUVECs were lysed in lysis buffer, and the samples were run on SDS/polyacrylamide gel electrophoresis using gradient gel (Atto Co, Tokyo, Japan) followed by electrophoretic transfer onto nitrocellulose membranes. After the blots were incubated for 1 hour in the blocking agent Blocking One (Nacalai Tesque), they were incubated overnight with anti-VEGFR2^23^, anti-Neuropilin1 (R&D Systems), anti-VEGFR2 phospho-Tyr951 (Cell Signaling), or anti-VEGFR2 phospho-Tyr1175 (Cell Signaling) at 4°C. Anti-rat or goat or rabbit IgG antibodies conjugated with horseradish peroxidase (HRP) were used as secondary antibodies (1:10000). A Can Get Signal Immunoreaction Enhancer solution kit (Toyobo, Osaka, Japan) was used for signal enhancement. Immunoreactivity was detected with the Chemi-Lumi One enhanced chemiluminescence kit (Nacalai Tesque). Signal intensity was calculated with Scion Image software (Scion Corp).

### Immunohistochemistry

Six-μm sections of tumors were fixed with 4% paraformaldehyde, washed twice in PBS, and blocked by 1% skim milk (BD Biosciences). Samples were incubated overnight with anti-CD31 antibody (BD Biosciences) and anti-VE-cadherin antibody (BD Biosciences) at 4°C. For immunofluorescent staining, anti-rat IgG antibodies conjugated with Alexa488 (Invitrogen) or Alexa546 (Invitrogen) were used as secondary antibodies. Nuclei were visualized with DAPI (Invitrogen). Stained cells were photographed with an inverted fluorescent microscope (BZ-9000, Keyence, Osaka, Japan).

### FACS analysis

Tumors were minced and then treated with dispase II (2.4 U/ml) and collagenase (1 mg/ml). The samples were incubated with 0.1% Trypsin/EDTA and added DNase I. Dissociated tumor cells were stained with combinations of PE-conjugated CD31 antibody MoAb (BD Biosciences) and FITC-conjugated anti-CD45 MoAb (BD Biosciences) and then subjected to analysis or purified endothelial cells using FACS Aria and FACS Canto (Becton Dickinson).

### RNA isolation and quantitative reverse transcription polymerase chain reaction (qPCR)

Total RNA was isolated from cells in HUVEC and undifferentiated ES cells using RNeasy (QIAGEN, Valencia, CA), according to the manufacturer's instructions. Reverse-transcription was performed with the SuperScript III first-strand synthesis system (Invitrogen). qPCR was performed using Power SYBR Green PCR Master Mix (Applied Biosystems, San Diego, CA) and a 7300 Real time PCR system (Applied Biosystems). The amount of target RNA was determined from the appropriate standard curve and normalized relative to the amount of *Gapdh* mRNA. Primer sequences are shown in supplementary Table S1.

### Animals

The present study was conducted in accordance with the guidelines for Animal Experiments of Hoshi University, which conform to the Guide for the Care and Use of Laboratory Animals in Japan. The animal experiments were approved in Hoshi University (Acceptance number; 13-001). The experiments were performed on C57BL/6J mice (Tokyo Laboratory Animals Science, Tokyo, Japan) and KOR-KO mice (The Jackson Laboratory, Bar Harbor, Maine, USA). The animals were normally fed standard laboratory food and water and housed in temperature (23 ± 1°C)-controlled rooms under a 12 h/12 h light/dark cycle.

### Graft tumor growth assay

The animals were anesthetized intraperitoneally with 70 mg/kg pentobarbital sodium (Nacalai Tesque, Kyoto, Japan). LLC or B16 cells were detached by trypsinization, collected, and counted. Cells were resuspended in a mixture of ECM gel (Sigma-Aldrich Japan K.K. Tokyo, Japan) and HBSS (ratio 3:1) at a concentration of 2 × 10^6^ cells/0.5 mL, and 0.5 mL of this suspension was then inoculated subcutaneously into the right lower back of mice. Tumor size was measured using a caliper and tumor volume was calculated as (L × W^2^)/2, where L = length and W = width. The tumor weight was measured after mice were sacrificed.

Two series of experiments were conducted. First, the effect of the global deletion of KOR was determined in KOR-KO mice and controls (C57BL6/6J mice). Second, the anti-tumor effect of the repeated administration of TRK-820, a KOR agonist, was investigated using three groups of C57BL/6J mice and KOR-KO mice. Two days after tumor implantation, mice were injected with either 0.9% saline or TRK-820 (0.1, 1, or 10 μg/kg, b.i.d.) every 12 hours for 12 days. For immunohistochemical studies, tumors were placed in optimum cutting temperature (OCT) compound media (Tissue-Tek; Sakura Finetechnical, Tokyo, Japan) and stored at −80°C until use.

### Statistical analysis

At least three independent experiments were performed. The data were subjected to a statistical analysis with one-way ANOVA. p < 0.05 was considered significant. Values are reported as means ± SEM.

## Author Contributions

K.Y., S.F., Y.H., A.Y., N.K., M.N., K.D., and S.K. carried out the experiments. K.Y., S.F., H.N., J.K.Y., and M.N. supervised the project. K.Y. and M.N. wrote the manuscript with inputs from all authors. All authors reviewed the manuscript.

## Supplementary Material

Supplementary Information Supplemental data set
