# Supplementary material for: к Opioids inhibit tumor angiogenesis by suppressing VEGF signaling
Source: Sci Rep. 2013 Nov 14;3:3213. doi: 10.1038/srep03213 (PMC3827603; doi:10.1038/srep03213)
Supplement: Supplementary Information — Supplemental data set [file srep03213-s1.doc]

**Scientific Reports**

**Supplementary information’s for**

**κ Opioids inhibit tumor angiogenesis by suppressing VEGF signaling**

Kohei Yamamizu, Sadayoshi Furuta, Yusuke Hamada, Akira Yamashita, Naoko Kuzumaki, Michiko Narita, Kento Doi, Shiori Katayama, Hiroshi Nagase, Jun K. Yamashita and Minoru Narita

This file includes:

Figure S1-S5

Table S1


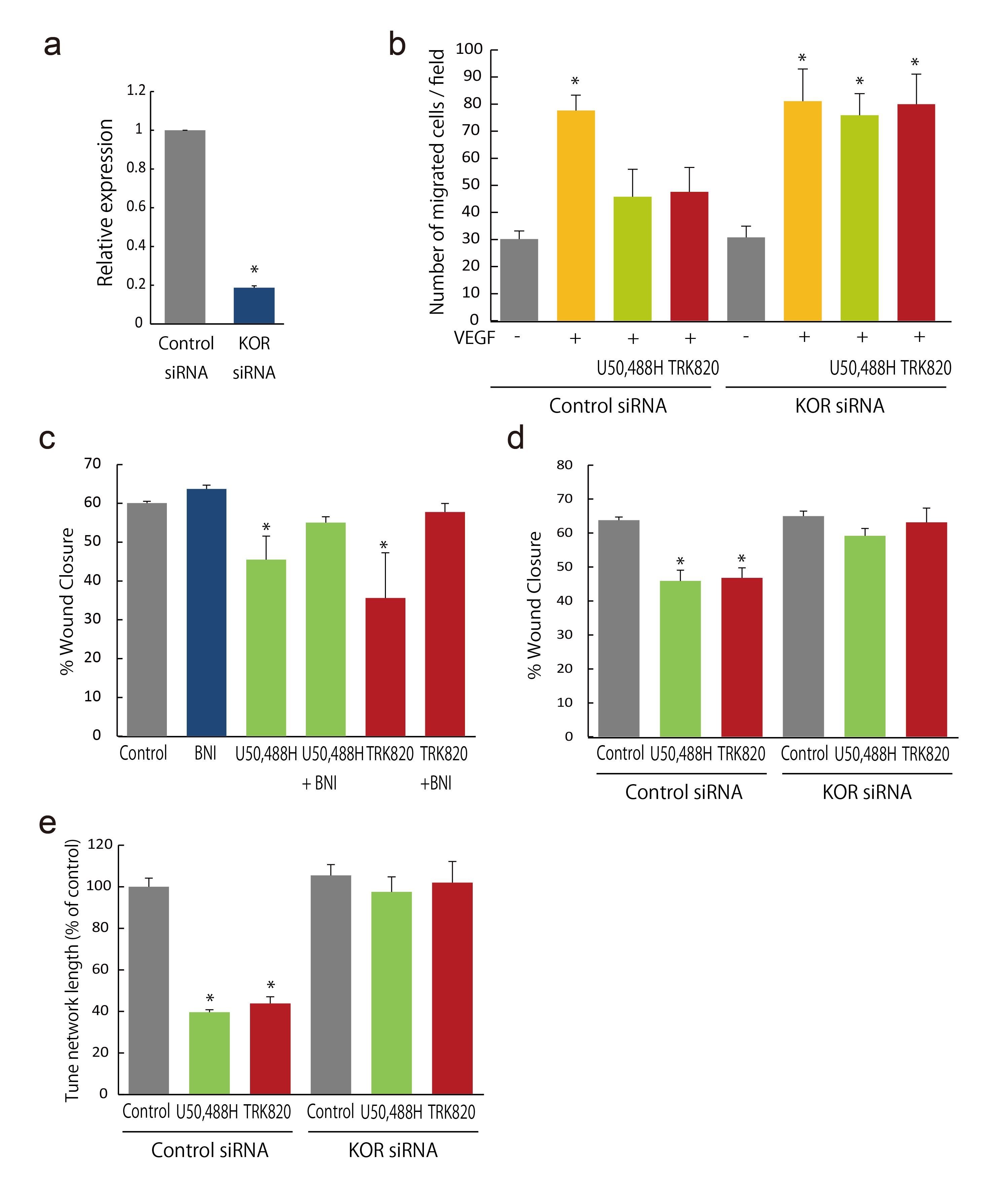


**Supplementary Figure 1. Inhibitory effects of KOR agonists, U50,488H and TRK-820, are restored by KOR antagonists, BNI, or knockdown of KOR with siRNA.**

(a) qPCR showing mRNA expression of KOR with control siRNA or KOR siRNA in HUVECs. (b) The boyden chamber assay. Inhibition of VEGF-induced chemotaxis was assessed after including U50,488H (10 μM), or TRK820 (10 μM) with control siRNA or KOR siRNA (n = 3, *p<0.05 vs. Control). (c) The wound-healing assay. Quantitative evaluation of the effect of opioid receptor agonists, U50,488H (10 μM) and TRK-820 (10 μM), and KOR antagonists, BNI (10 μM) on HUVEC migration assay. Three independent experiments are shown (*p<0.05 vs. Control). (d) The wound-healing assay. Quantitative evaluation of the effect of opioid receptor agonists, U50,488H (10 μM) and TRK-820 (10 μM) with control siRNA or KOR siRNA (n = 3, *p<0.05 vs. Control). (e) HUVEC tube formation assay. Quantitative evaluation of the effect of opioid receptor agonists, U50,488H (10 μM) and TRK-820 (10 μM) with control siRNA or KOR siRNA (n = 3, *p<0.05 vs. Control).


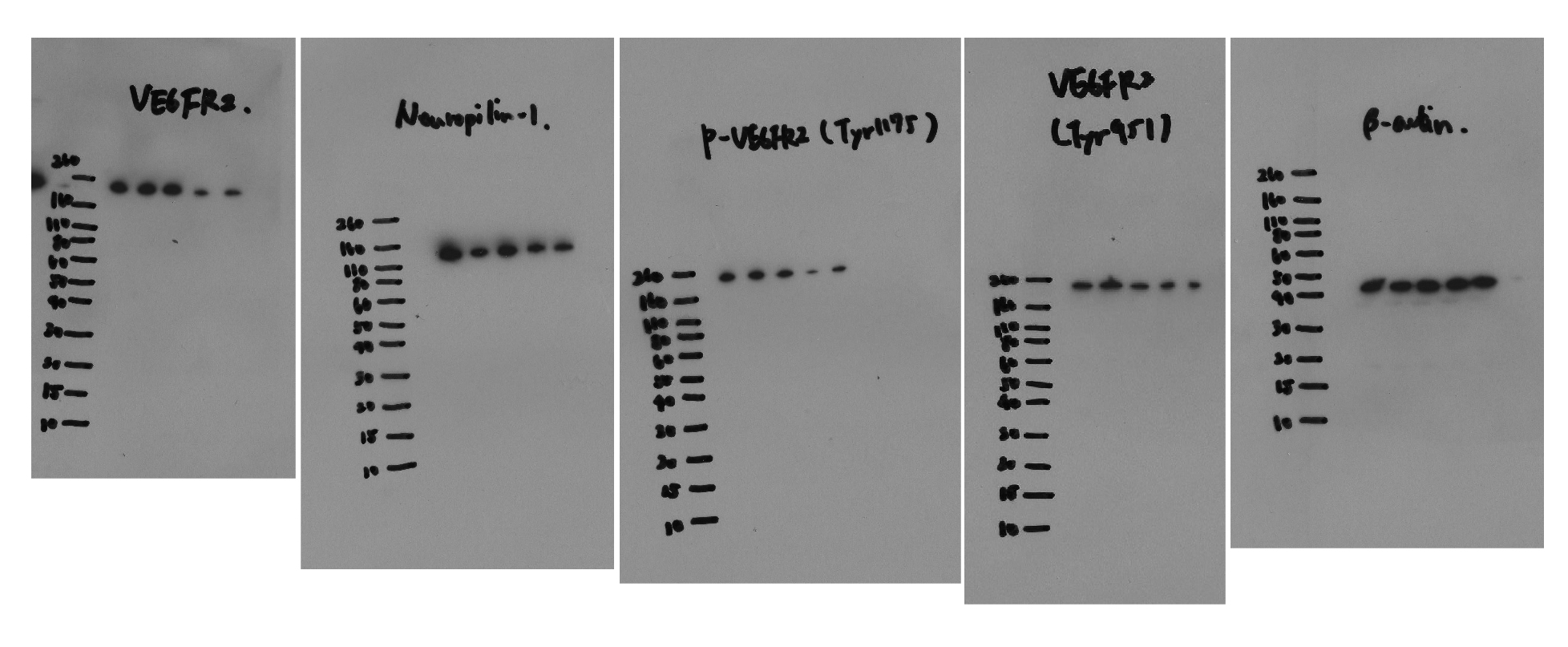


**Supplementary Figure 2. Inhibitory effects of KOR agonists, U50,488H and TRK820, on VEGFR2 expression in HUVECs.**

Western blotting of VEGFR2, Neuroplin1, VEGFR2 phospho-Tyr951, VEGFR2 phospho-Tyr1175, or β-actin after 24 hr culture with DAMGO (10 μM), SNC80 (10 μM), U50,488H (10 μM) or TRK820 (10 μM). These gels have been run under the same experimental conditions (see Methods). These cropped blots are used in the main figure (Figure 2) and these full-length blots are included in the supplemental figure.


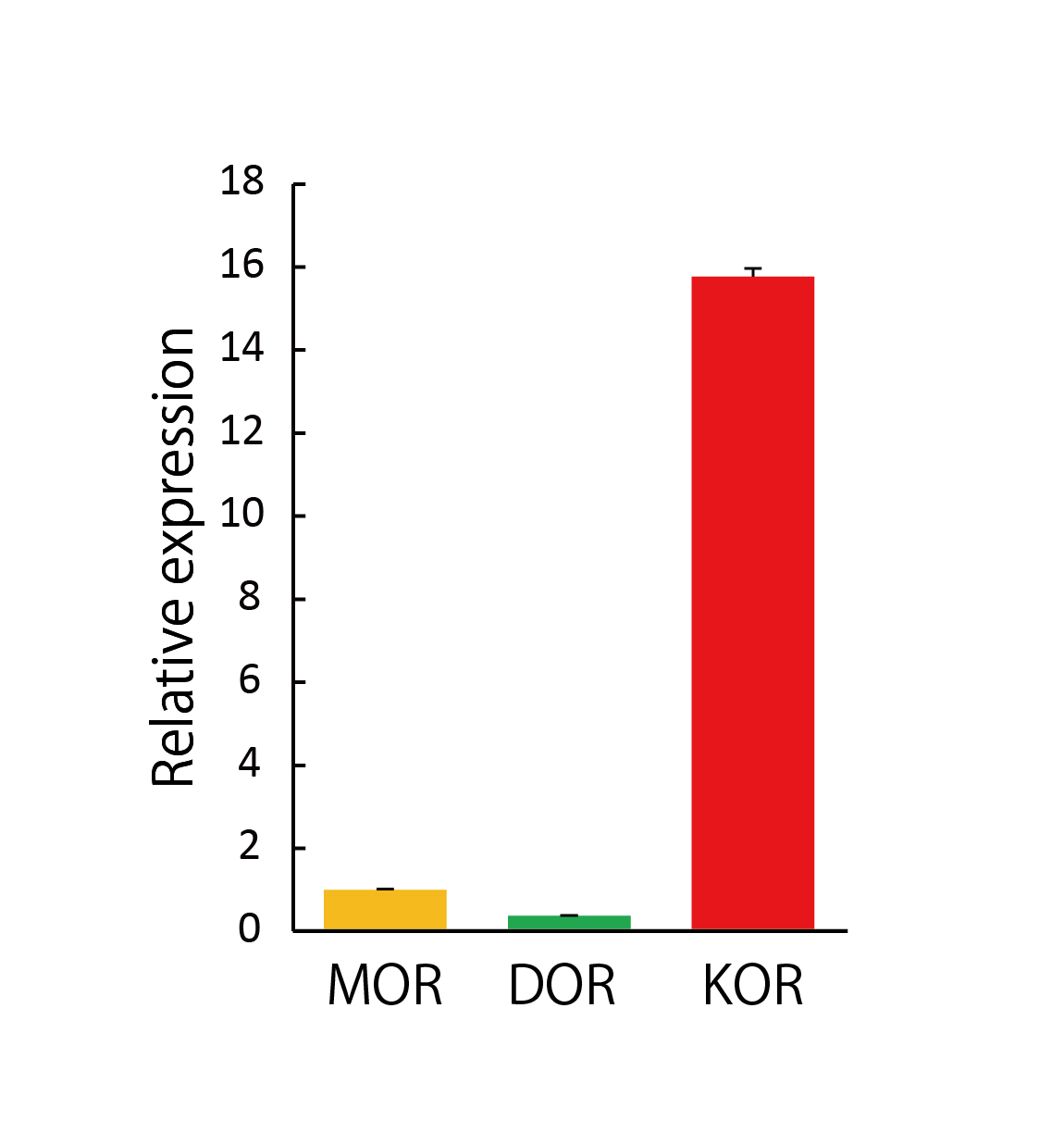


**Supplementary Figure 3. Expression of KOR in tumor endothelial cells.**

qPCR showing mRNA expression of MOR, DOR, and KOR in purified tumor endothelial cells from LLC grafted in control mice.


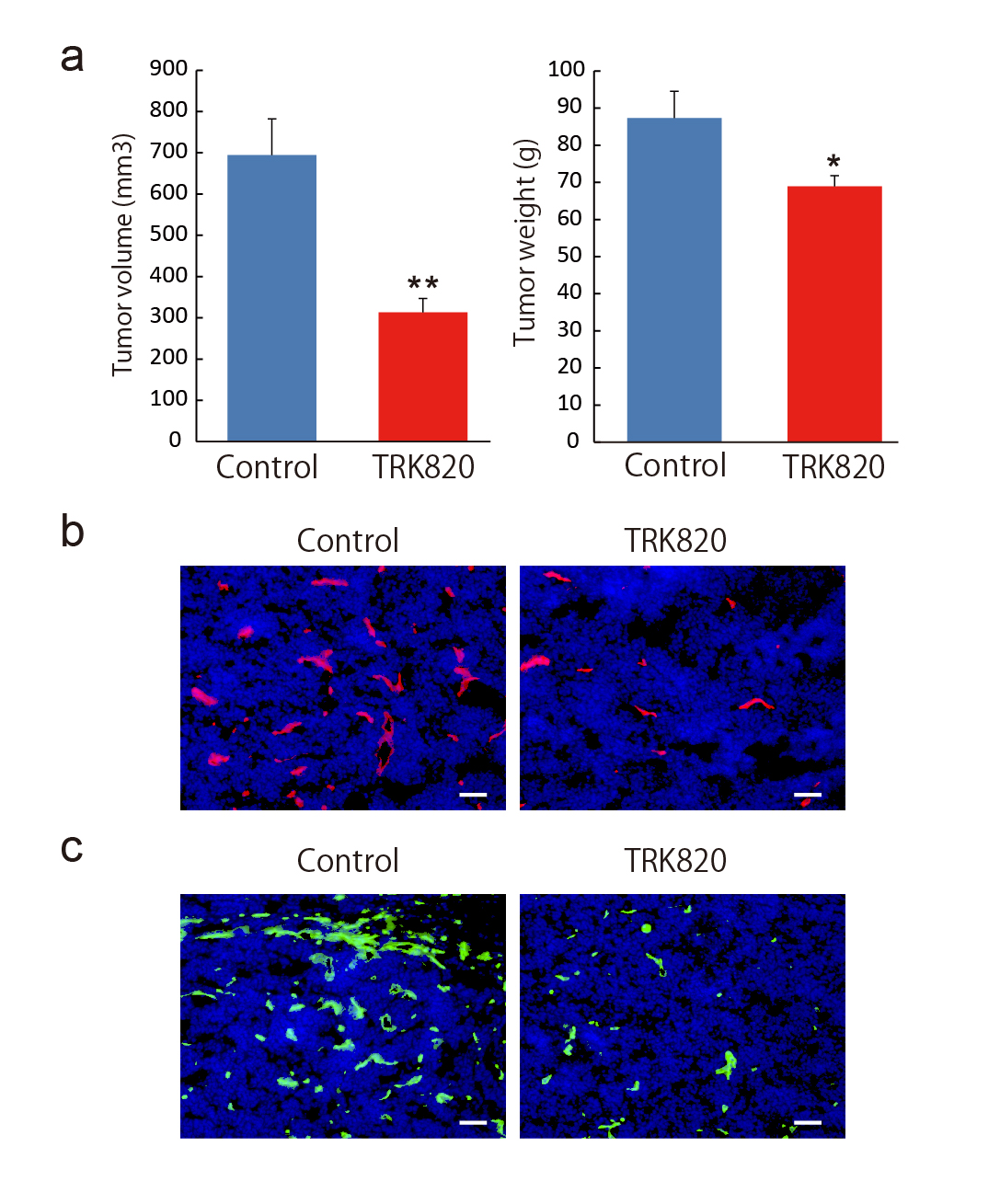


**Supplementary Figure 4. Suppression of tumor growth by a KOR agonist, TRK820 in xenograft mice at 7 days after tumor transplantation.**

(a) Quantitative analysis of tumor volume and tumor weight among PBS-treated (n = 8) and TRK820 (1 μg/kg)-treated (n = 8) mice at 7 days after tumor transplantation (**p<0.01, *p<0.05 vs. Control). (b) Fluorescent staining for CD31 (red) at 7 days after tumor transplantation. Nuclei are stained with DAPI (blue). Left panel, PBS treated. Right panel, TRK820 (1 mg/kg)-treated. Scale bars: 50 μm. (c) Fluorescent staining for VE-cadherin (green) at 7 days after tumor transplantation. Nuclei are stained with DAPI (blue). Left panel, PBS treated. Right panel, TRK820 (1 mg/kg)-treated. Scale bars: 50 μm.


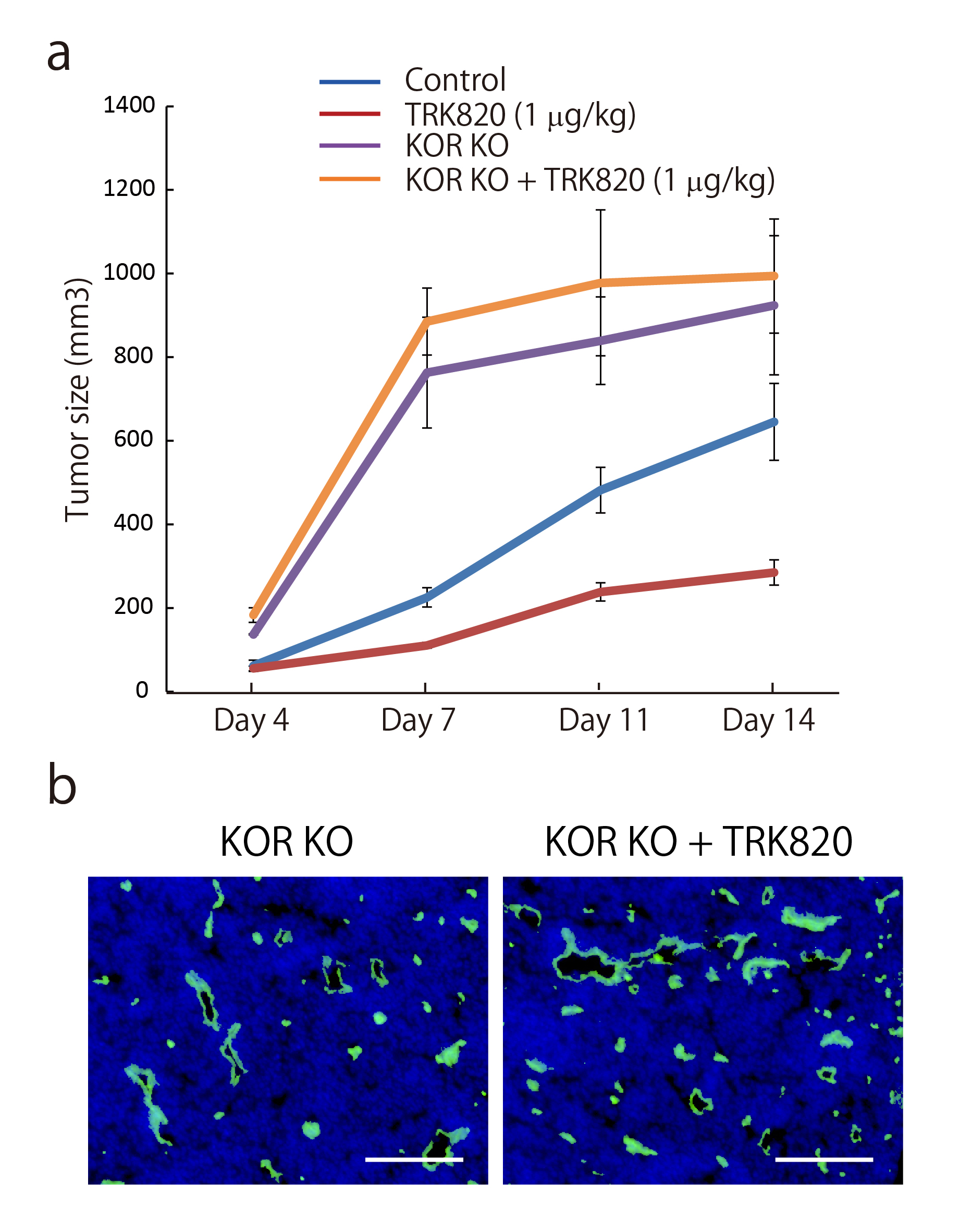


**Supplementary Figure 5. TRK820 specifically induced inhibition of the tumor growth through KOR receptors.**

(a) Quantitative analysis of tumor size among PBS-treated (n = 16) and TRK820 (0.1 μg/kg (n = 9), 1 μg/kg (n = 17), 10 μg/kg (n = 9)-treated control mice or KOR KO mice at 4, 7, 11, 14 days after tumor transplantation. (b) Fluorescent staining for VE-cadherin (green) at 14 days after tumor transplantation. Nuclei are stained with DAPI (blue). Left panel, PBS treated KOR KO mice. Right panel, TRK820 (1 mg/kg)-treated KOR KO mice. Scale bars: 200 μm.

Supplementary Table 1: Primer list for RT-PCR

| Gene | Sequence |
| --- | --- |
| MOR Forward  Reverse | AGA CTG CCA CCA ACA TCT ACA T |
|  | TGG ACC CCT GCC TGT ATT TTG |
| DOR Forward  Reverse | GCT GTG CTC TCC ATT GAC TAC |
|  | GAT GTC CAC CAG CGT CCA GAC |
| KOR Forward  Reverse | AGT CCC CCA TTC AGA TCT TCC |
|  | ACA GCA ATG TAG CGG TCC AC |
| VEGFR2 Forward  Reverse | TGA TCG GAA ATG ACA TGG A  CAC GAC TCC ATG TTG GTC AC |
| Neuropilin1 Forward  Reverse | TGT GAA GTG GAA GCC CCT AC  CAC CTG TGA GCT GGA AGT CA |
| GAPDH Forward  Reverse | CCC ACG GCA AGT TCA ACG G  CTT TCC AGA GGG GCC ATC CA |

**Methods**

**KOR knockdown using siRNA**

siRNA targeting human KOR were purchased from invitrogen (Stealth RNAi). Stealth RNAi for KOR (10 nM) or control (10 nM) were transfected HUVEC using Lipofectamine RNAiMAX (invitrogen) according to the manufacture’s instruction. After 2 days, HUVEC were examined by a boyden chamber assay, a wound-healing assay, and a tube formation assay.
